# Detection of atypical network development patterns in children with autism spectrum disorder using magnetoencephalography

**DOI:** 10.1371/journal.pone.0184422

**Published:** 2017-09-08

**Authors:** Fang Duan, Katsumi Watanabe, Yuko Yoshimura, Mitsuru Kikuchi, Yoshio Minabe, Kazuyuki Aihara

**Affiliations:** 1 Institute of Industrial Science, the University of Tokyo, Tokyo, Japan; 2 Research Center for Advanced Science and Technology, the University of Tokyo, Tokyo, Japan; 3 Department of Intermedia Art and Science, Waseda University, Tokyo, Japan; 4 Research Center for Child Mental Development, Graduate School of Medical Science, Kanazawa University, Kanazawa, Japan; Universitatsklinikum Tubingen, GERMANY

## Abstract

Autism spectrum disorder (ASD) is a developmental disorder that involves developmental delays. It has been hypothesized that aberrant neural connectivity in ASD may cause atypical brain network development. Brain graphs not only describe the differences in brain networks between clinical and control groups, but also provide information about network development within each group. In the present study, graph indices of brain networks were estimated in children with ASD and in typically developing (TD) children using magnetoencephalography performed while the children viewed a cartoon video. We examined brain graphs from a developmental point of view, and compared the networks between children with ASD and TD children. Network development patterns (NDPs) were assessed by examining the association between the graph indices and the raw scores on the achievement scale or the age of the children. The ASD and TD groups exhibited different NDPs at both network and nodal levels. In the left frontal areas, the nodal degree and efficiency of the ASD group were negatively correlated with the achievement scores. Reduced network connections were observed in the temporal and posterior areas of TD children. These results suggested that the atypical network developmental trajectory in children with ASD is associated with the development score rather than age.

## Introduction

Autism spectrum disorders (ASD) are pervasive developmental disorders characterized by impaired social abilities and restricted and repetitive patterns of activity. According to the Diagnostic and Statistical Manual of Mental Disorders, Fourth Edition (DSM-IV) [1], symptoms of ASD must be present before 3 years of age. Usually, parents of children with ASD notice abnormal signs in the first 2 years of life [2]. However, the Diagnostic and Statistical Manual of Mental Disorders, Fifth Edition (DSM-5) revised the diagnostic criteria for ASD in several aspects [3], including one change suggesting that the onset of symptoms in childhood may not manifest because of limited capacity and learned strategies. This change has contributed to increased ASD research being conducted in children. The new criteria also include other changes, e.g. the revision of language impairments, and the addition of sensory problems into “unusual activities.” It is important to note that parents often notice difficulties in sensory modulation as the first symptom in children with ASD [4].

Brain graphs have recently become commonly used in neuroscience [5–12]. The graph theory provides a quantitative way to analyze the properties of brain connectivity [13, 14]. Brain graphs simply model complicated brain networks by building a set of nodes and edges between them. The function and performance of the brain could be subsequently elucidated by studying the topological organization of brain graphs [10]. The integration of neuroscience and graph theory has produced novel findings. These studies have focused mainly on comparisons of graph indices between clinical and control groups [6, 15–19]. The nodes of brain graphs have been defined through recording sensors or anatomical regions, while the edges indicate the associations between the nodes. ASD is a developmental disorder that involves changes in many brain areas [20]. Frontocortical overgrowth at early stages in ASD has been reported in a structural study, with overgrowth no longer apparent after the age of approximately 7 years [21]. The frontal and temporal lobes have been found to be more affected than the parietal and occipital lobes at an early age in ASD [22]. The trajectory of neurodevelopment in ASD is aberrant. Brain network characteristics are related to topological changes in the graph during development [10]. Therefore, studies of brain graphs in patients with ASD should consider network development, i.e., how the network changes during the development of participants. In our previous magnetoencephalography (MEG) study, a negative correlation between cognitive performance and the small-world brain functional networks was observed in typically developing (TD) children while they watched a video [23]. MEG, a non-invasive and real-time functional neuroimaging technique, can record magnetic fields produced by the electrical currents of neuronal spikes through sensitive magnetometers [24]. Since it is a child friendly technique [25], neural activity can be measured using MEG on a timescale of milliseconds, and shorter recording sessions are required than those required for functional magnetic resonance imaging (fMRI), which works on a timescale of seconds. MEG data can also be recorded without wearing a cap requiring electrode cream which may cause children to feel uncomfortable during electroencephalography (EEG) recording. Moreover, the reference-free property of MEG makes it an ideal tool for measuring the functional connectivity between regions [26]. Several studies have reported that functional connectivity is associated with development in children with ASD [27, 28]. Therefore, we hypothesized that brain network development in children with ASD can also be observed using the graph theory.

We aimed to describe the atypical development of children with ASD, using graph theory. In our previous studies of TD children, the network pattern revealed by brain graph indices demonstrated that graph indices were related to cognitive performance [23]. In the present study, we considered the relationship between graph indices and indicators of development as the network development pattern (NDP) of the brain. Raw scores on the achievement scale, a type of cognitive performance scale in the Kaufman Assessment Battery for Children (K-ABC) [29], and the ages of the children were used as indicators of development. We examined the networks in children with ASD and TD children, and compared the NDPs between the two groups. Data acquisition and network estimation methods for the ASD group were the same as for the TD group and have been previously published [23]. Network level indices were analyzed using the Watts-Strogatz model, the so-called small-world model [30]. Using two main indices of Watts-Strogatz model, the local information processing ability and the global efficiency for integrating and transferring information were evaluated. The changes in the local and global balance of the brain network during development can be understood though the NDPs of small-world indices. The NDPs for specific regions were identified using nodal level indices, namely the nodal degree and nodal efficiency [31]. The nodal degree indicates the importance of the sensor or node to the entire graph. The nodal efficiency reflected the integrated processing ability and information flow of nodes. The NDPs at the level of nodes demonstrate that the importance of the node varies during development. Our findings indicate that the NDPs of children with ASD were different from TD children, and implied that the network developmental trajectories of children with ASD and TD children were different.

## Methods

### Participants

Participants in the clinical group included 28 children (23 boys, 5 girls) diagnosed with ASD, who were recruited from Kanazawa University Hospital and the prefectural hospitals in Toyama. The children with ASD had a mean age of 66.2 months (range 40–93 months). The diagnosis of individuals in the clinical group was made by a clinical psychiatrist and a speech language hearing therapist with 5 years of experience of working with ASD. The Autism Diagnostic Observational Schedule, Generic [32], Diagnostic Interview for Social and Communication Disorders [33], and the DSM-IV were used for diagnosis at the time of the MEG recording. Participants in the control group were 30 TD children (25 boys, 5 girls) with no psychiatric history. The TD children had a mean age of 63.9 months (range 39–81 months). There was no significant difference in age between the two groups (*p* = 0.4750, Student’s t-test). All participants had normal hearing and visual acuity according to their available medical records, i.e., they had not been noted as having any problems with hearing or visual acuity at the three-year-old routine health checkup, and they demonstrated no problems with hearing or visual acuity in their daily lives.

Before enrolling in the clinical trial, the following information was given to the parents of each potential participant: (1) research participants have the right to refuse treatment and will not lose any benefits to which they are entitled, and (2) research participants may choose to cease participation in the clinical trial at any time without losing the benefits to which they are entitled. The parents consented for their children to participate in the study with full knowledge of the experimental nature of the research. Written informed consent was obtained prior to participation in the study. The study was conducted in accordance with the Declaration of Helsinki. The Ethics Committee of Kanazawa University Hospital and the Ethics Committee of The University of Tokyo approved the methods and procedures that were used in this study.

The Japanese adaptation of the K-ABC was performed with all children [29]. The K-ABC and MEG recordings were performed on separate days. The raw scores on the achievement scale of the K-ABC were used as an index of development. The achievement scale evaluated the crystallized intelligence and quantitative reasoning ability according to the Cattell-Horn-Carroll model [34]. We obtained the achievement scores from the Expressive Vocabulary, Arithmetic, and Riddles sub-tests on the K-ABC. The Expressive Vocabulary sub-test required children to provide the names of familiar objects in photographs. The Arithmetic sub-test required the children to solve arithmetic problems within the context of a story. The Riddles sub-test required the children to retrieve a word from hints relevant to the word. The raw scores of the Expressive Vocabulary and Arithmetic sub-tests did not differ between the groups (*p* = 0.5052 and *p* = 0.7690, respectively). The scores on the Riddle sub-test differed slightly but not significantly between the groups (*p* = 0.1181). The achievement score was the sum of the scores of the three sub-tests. The means ± standard deviations of the achievement scores were 37.9 ± 16.5 and 41.4 ± 11.3 in the ASD and TD groups, respectively. The achievement scores were not significantly different between the groups (*p* = 0.3516). Table 1 summarizes the characteristics of the participants including the sex, age, sub-tests score, and achievement scale.

**Table 1:** Participants’ characteristics (sex, age, scores on sub-tests, and achievement scale).

Group	ASD	TD	*p*-value	Cohen's *d*
Sex(Boys/Girls)	23/5	25/5		
Age (M ± SD)	66.2 ± 12.0	63.9 ± 11.8	0.4750	0.19
Expressive vocabulary (M ± SD)	17.0 ± 6.3	17.9 ± 3.6	0.5052	0.18
Arithmetic (M ± SD)	13.1 ± 6.6	13.6 ± 5.2	0.7690	0.08
Riddle (M ± SD)	7.8 ± 6.0	9.8 ± 4.0	0.1181	0.39
Achievement scale (M ± SD)	37.9 ± 16.5	41.4 ± 11.3	0.3516	0.25

ASD, autism spectrum disorder; TD, typically developing; M, mean; SD, standard deviation.

### MEG recordings and preprocessing

MEG recordings and data preprocessing were performed according to a previously described method [23]. A custom-made MEG system for children (PQ 1151R; Yokogawa/KIT Corp, Kanazawa, Japan) with a 151-channel whole-head coaxial gradiometer was used to record the data [27] at a sampling rate of 1000 Hz in a shielded room (Daido Steel, Nagoya, Japan). We introduced the participants to the MEG environment on the day that they completed the K-ABC test. The MEG recording sessions were subsequently performed on the second day. Several videos of children programs were prepared for the MEG recording. Each child selected a program according to his or her preference for use during the MEG recording. Tokusatsu videos (i.e., live-action films) were chosen by 17 children. More than half of the children (n = 34) chose animations during the MEG recording. Six children selected other types of animated video programs. A video program with static pictures was selected by one child with ASD. The names of the children programs are listed in S1 Table. At the time of the recording session, the participants were escorted to the shielded room. During the MEG recording sessions, the participants lay comfortably on a bed with their head inside the MEG system helmet. The video program was projected onto a screen and participants heard binaural audio through a tube. During the recording, a staff member remained in the shielded room to comfort and encourage the participant as necessary. The staff member also confirmed that participants were enjoying the videos during each session. MEG data were recorded for 3 min during each session. Data from both the ASD and TD groups have been previously reported as a part of our related research [26]. Data from the TD group were also reported in our previous study of healthy children [23].

Data preprocessing was performed using MATLAB (The MathWorks, Inc., Natick, MA, USA) in conjunction with the FieldTrip toolbox [35] and the EEGLAB toolbox [36]. The recorded MEG data were filtered by a 0.1–100 Hz band-pass filter. Artifacts were removed from the MEG data using independent component analysis with the FastICA algorithm from the FieldTrip toolbox. The independent components with extremely high kurtosis, i.e., the fourth-order cumulant, were automatically removed as artifacts. After artifacts were removed, the MEG data were resampled at 250 Hz. The epochs from 40–60 s were selected for analysis, when data qualities were similar between the participants and were merely affected by fatigue and distraction.

### Brain graph construction

In a similar manner to conventional methods, we considered the MEG recording sensors as the nodes of the graph. To generate the edges of the graph, the associations between the nodes needed to be determined. For each participant, the association matrix of the nodes was computed using Mutual Information, which is a measure sensitive to both linear and nonlinear interactions [37]. The element *a*_*ij*_ of the association matrix was calculated as follows:

$$a_{ij}=\sum_{x_{i}}\sum_{x_{j}}p(x_{i},x_{j})\text{log}(\frac{p(x_{i},x_{j})}{p_{x_{i}}(x_{i})p_{x_{j}}(x_{j})}),$$ (1)

where *x*_*i*_ and *x*_*j*_ are the variables of sensors *i* and *j*, respectively, and *p*(·) represents the probability density function. We estimated the probability density functions by binning the coordinate axes of the variables. The present study adopted the criterion of outlier elimination during the estimation of density functions as either *x* or *y* having a value with a probability of 0.5% or less (bilateral). The association matrices of the entire band of MEG data were also estimated.

We determined the edges of the brain graph by thresholding the association matrix. The element of the binary adjacency matrix was defined as follows:

$$A_{ij}=\left\{ \begin{array}{l} \text{1}\\ \text{0} \end{array}\right.\;\begin{array}{c} when\;a_{ij}\geq T,\\ otherwise, \end{array}$$ (2)

where *T* is the binariztion threshold for. A non-zero element in the adjacency matrix corresponds to an edge between two nodes. The threshold values were set individually to ensure that the brain graph of each child had an equal number of edges, following standard protocols from the literature [37, 38], to allow for simplified comparisons across networks. A symmetric association matrix will generate a symmetric binary adjacency matrix. Therefore, we considered the brain networks to be undirected graphs with equal weights. The targeted number of edges was varied for analysis.

### Network evaluation

We focused on the graph indices at the level of both the network and the node. Network level indices included the clustering coefficient and the characteristic path length, which are common indices for undirected graphs with equal weights in the small-world network theory [30]. Nodal level indices included the nodal degree and nodal efficiency, which performs well in identifying special nodes of the brain network [31].

The network level indices, including the clustering coefficient (*C*) and the characteristic path length (*L*), can indicate the efficiency of information transfer in the network. In a network with *N* nodes and *K* edges, *C* is defined as follows:

$$C=\frac{2}{N}\sum_{i}\frac{\sum_{j}\sum_{m=j+1}^{N}A_{ij}A_{jm}A_{mi}}{k_{i}(k_{i}-1)},$$ (3)

where *k*_*i*_ is the degree, i.e., the number of edges incident to node *i*, and *C* is the probability that two edges of a node are the edges of a subgraph. A graph with a high *C* value indicates that the graph may have strong local connections for information processing. A regular lattice is a typical graph structure with a high *C* value. The *L* of the network is defined as follows:

$$L=\frac{2}{N(N-1)}\sum_{i}\sum_{j=i+1}^{N}d_{ij},$$ (4)

where *d*_*ij*_ is the shortest path length between nodes *i* and *j*, and *L* is the average of the shortest paths connecting any two nodes on the graph. A short *L* indicated that the graph holds sufficient long-distance connections for information transfer. A graph with a random structure usually possesses a short *L*. The small-world network graph is a graph structure that has the combined properties of both a random graph and a regular graph. A regular lattice with a proper portion of randomly re-wired edges can generate a small-world network, which exhibits an optimal balance of global and local efficiency.

The nodal level indices, such as the nodal degree and nodal efficiency, can indicate the importance and efficiency of information processing in a node of the graph. The nodal degree is the number of neighbors that a node has. The number of edges connected to the node is equal to the degree, and is defined as follows:

$$k_{i}=\sum_{i\neq j}A_{ij}.$$ (5)

A node with a very high degree is usually considered to be a hub, which largely contributes to information spreading and network stability in the graph. By observing the changes of nodal degrees during development, the developmental importance of the nodes can be explicit. The nodal efficiency is defined as the mean of the reciprocal of the shortest path length as follows:

$$e_{i}=\frac{1}{N-1}\sum_{i\neq j}\frac{1}{d_{ij}}.$$ (6)

The nodal efficiency evaluates the capacity of each node to process and propagate information through the entire graph. The values of the nodal efficiency range between 0, i.e., a node without any neighbors, and 1, i.e., a node that connects to all the other nodes. The developmental trajectory of nodal efficiency indicates the variation in the integrated processing ability and reflects the quantitative change in the information flow of the node.

### Statistical analysis

We performed the Shapiro-Wilk test to check the normality of graph indices. The null-hypothesis of the Shapiro-Wilk test is that the samples come from a normal distribution with unspecified mean and variance. The Shapiro-Wilk test was performed on the mutual information of all 11325 possible pairs of channels, *L* and *C* for the average degree from 10 to 40, and nodal degree and efficiency for all 151 channels for the average degree of 23 in TD, ASD, and all groups, respectively.

The two-sided Wilcoxon rank sum test was performed to determine if the medians of the graph indices of the two groups were equal. The two-sided Wilcoxon rank sum test was also performed on the mutual information, *L* and *C*, and the nodal degree and nodal efficiency at an average degree of 23, to determine potential significant differences between the ASD and TD groups.

Pearson correlation coefficients were calculated between the graph indices and the development indices for the ASD and TD groups separately. Here, the graph indices included the *L* and *C* for the average degree from 10 to 40, and the nodal degree and nodal efficiency of all 151 channels for the average degree of 23. The developmental indices were age and achievement scores. The *p*-value of each correlation was also estimated. We considered significant correlations with *p*-values less than 0.05 as NDPs.

## Results

### Normality of graph indices

The results of the Shapiro-Wilk test indicated that a considerable portion of the indices showed non-Gaussian distributions at a significance level of 0.05. For the elements of the association matrices with weak associations, the null hypothesis that the mutual information between the channels was normally distributed, with unspecified mean and variance, was rejected. The associations with high mutual information could mainly be considered as being from normal distributions. The network level indices could be considered normally distributed when the average degree was approximately 20. The normality of network level indices may relate to a proper network density for parametric analysis. Most of the indices at the level of the nodes did not come from normal distributions at a significance level of 0.05. Compared with the TD group and all participants, the normality of nodal level indices in the ASD group was more common. The nodal efficiencies for the 96 channels of the ASD group were considered to be normally distributed when the average degree was 23. Due to the strong non-Gaussian distributions of graph indices, we chose the Wilcoxon rank sum test to determine the difference between TD children and children with ASD. The Wilcoxon rank sum test does not require the assumption of normal distributions.

### Association matrices and networks in children

**Fig 1:**
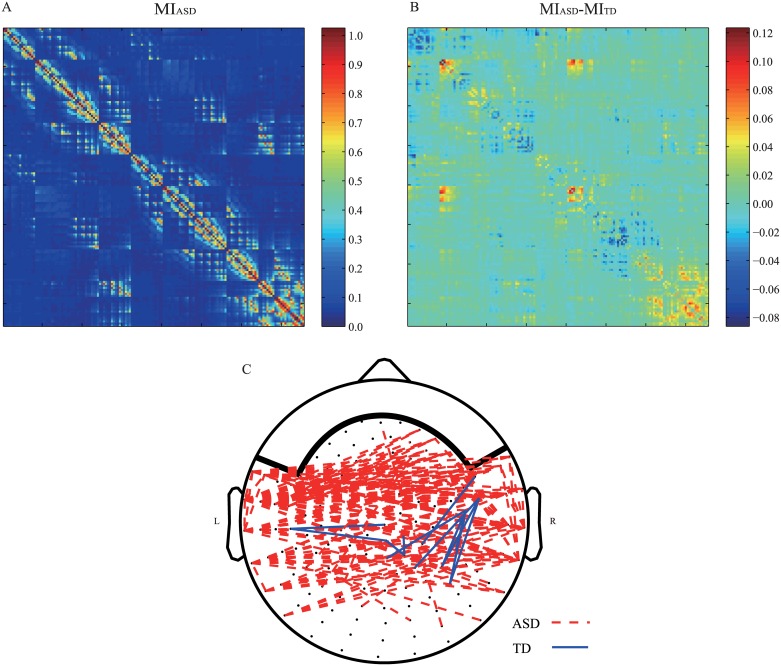
Association matrix and difference between groups. (A) The average association matrix in children with ASD. (B) Subtraction of the average association matrix in TD children from that in the children with ASD. (C) The connections showing significantly different (*p* < 0.05) mutual information between the ASD and TD groups.

Fig 1A shows the average of the association matrix for children with ASD. Fig 1B shows the difference between the association matrices for children with ASD and TD children. The significantly different connections are shown in Fig 1C. The red dashed line indicates that the connection in the ASD group is stronger than in the TD group, and the blue solid line corresponds to a stronger connection in the TD group. The results show that the connection in the ASD group is stronger in the bilateral temporal lobes. Several stronger associations in TD children are located in the anterior-medial and the medial-posterior areas.

**Fig 2:**
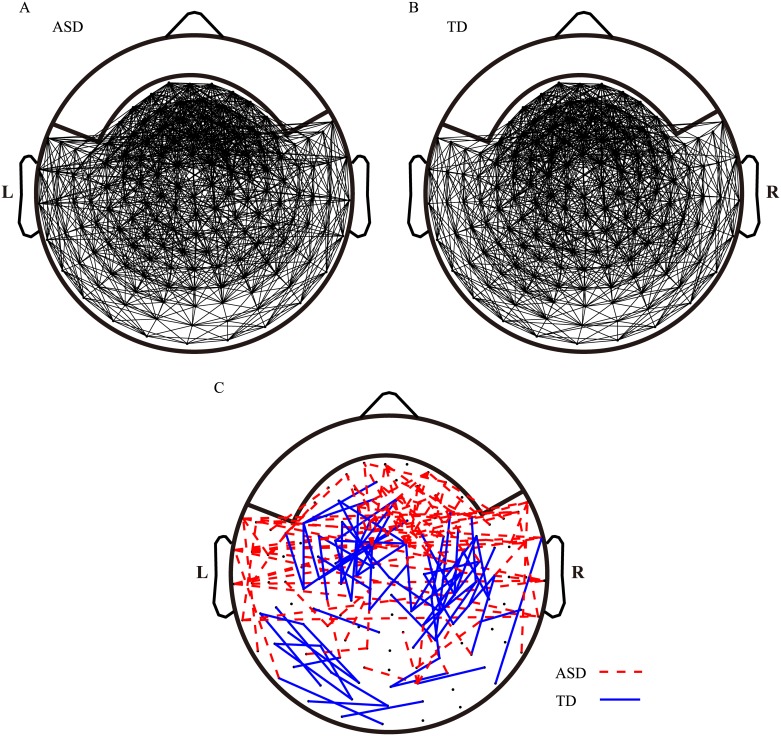
Mean brain networks of the two groups. The mean brain networks of (A) children with ASD and (B) TD children were generated by binarizing the mean association matrices to the adjacency matrices with an equal number of 1. The degree of (A) and (B) was 23. (C) The dashed lines indicate specific connections that exist only in the mean brain networks of children with ASD, and the solid lines indicate connections that exist only in the mean brain networks of TD children.

Fig 2 shows the mean brain networks in children with ASD and TD children. The threshold values were set to ensure that the degree of the mean network in each group was 23. This degree number, which was in the range that showed a prominent association between network level indices and development indices in children, was determined by manual inspection. Fig 2C shows the specific connections in children with ASD and TD children. The specific edges of the ASD group were long-range connections between the bilateral temporal lobes, the temporal and frontal lobes, and the temporal and parietal lobes. Local connections in the right frontal area were also observed. The specific edges of the TD group were mainly short-range connections between or within the left frontal and central areas, the right central and parietal areas, and the left temporal and occipital areas.

### Network level indices

**Fig 3:**
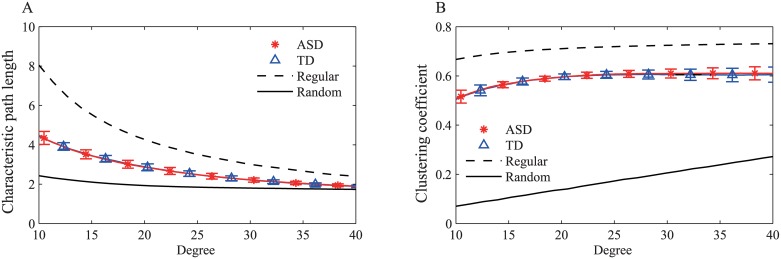
Network level indices as a function of degree. (A) The characteristic path length (*L*) and (B) the clustering coefficient (*C*) in children with ASD (asterisks) and TD children (triangles) as a function of the average degree of the network. The error bars correspond to standard deviations. The corresponding values of the regular and random networks are indicated by dashed and solid lines, respectively.

Fig 3 shows the results of the network level indices, *L* and *C*. The range for the degree of the network was selected based on the topologically interesting range found in our previous work [23]. There were no significant differences (*p* > 0.05) in the *L* or *C* between the children with ASD and the TD children. The *L* and *C* of both groups were between the values corresponding to the regular and random networks. The *L* was closer to the value of the random network than that of the regular network. The results of *C* showed that the *C*s in the participants’ brain networks were similar to those in the regular network. The network level indices of both the children with ASD and the TD children were somewhat indicative of small-world networks.

**Fig 4:**
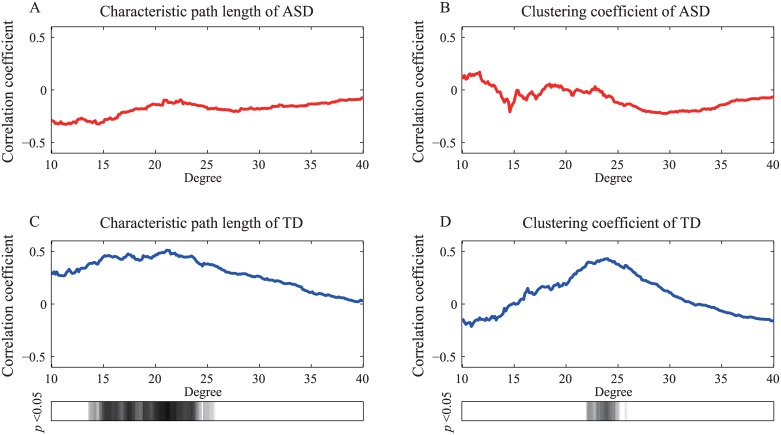
Correlation coefficients between network level indices and the participants’ age. Panels (A) and (B) show the correlation coefficients between *L* and *C*, respectively, as well as the age of the children in the ASD group as a function of the degree. The correlation coefficients between *L* and *C* and the age of the children in the TD group are shown in (C) and (D), respectively. The shaded bars show the intervals at which the graph indices and age were significantly correlated (*p* < 0.05).

**Fig 5:**
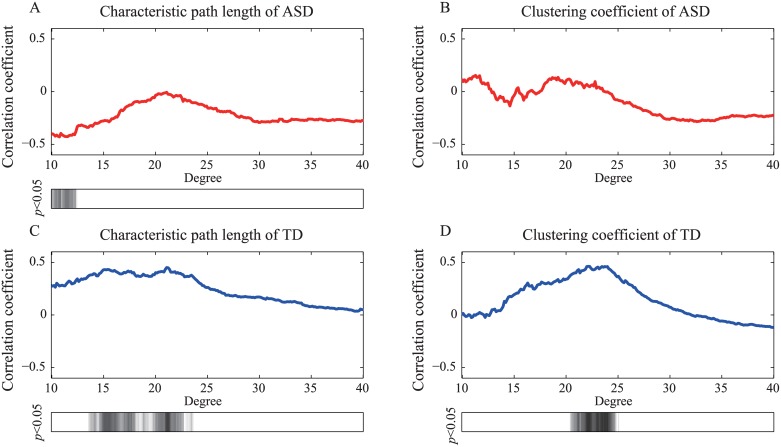
Correlation coefficients between network level indices and the achievement scores. The *L* and *C* results of ASD group are shown in panels (A) and (B). Panels (C) and (D) correspond to the results from the TD group. The significantly correlated intervals (*p* < 0.05) are also indicated by shaded bars.

In the network level analysis, the network level and development indices were significantly correlated in the TD group. The level of significance of the correlations in the ASD group was less than in the TD group. Fig 4 shows the correlation coefficients between the network level indices and the age of each group as a function of the degree. Fig 5 shows the correlation coefficients between the network level indices and the achievement scores. The *L* showed a weak (0.01 < *p* < 0.05) correlation with the achievement scores when the network degree was less than 12 in the ASD group. Both the *L* and *C* strongly correlated (*p* < 0.01) with age and achievement scores when the network degree was approximately 23 in the TD group.

### Nodal level indices

**Fig 6:**
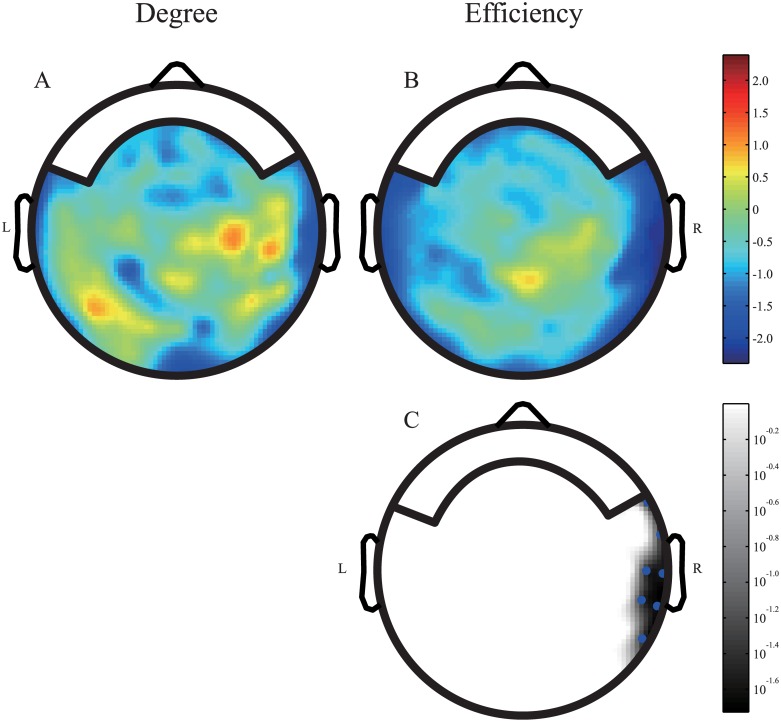
Comparison between nodal level indices in children with ASD and TD children. The network degree was set at 23. Panels (A) and (B) show the maps of the values of the z-statistic. Warm colors indicate high median values in the TD group, and the cool colors represent high median values in the ASD group. Panel (C) shows the *p*-value map showing significant differences (*p* < 0.05). Blue dots indicate the regions in which the *p*-values are between 0.01–0.05.

The nodal level indices were studied in order to examine the network patterns of specific regions. We did not find any significant difference (*p* > 0.05) in the nodal degree between the TD and ASD groups. Significant differences between the groups for the nodal efficiency were found in the right temporal area (Fig 6).

**Fig 7:**
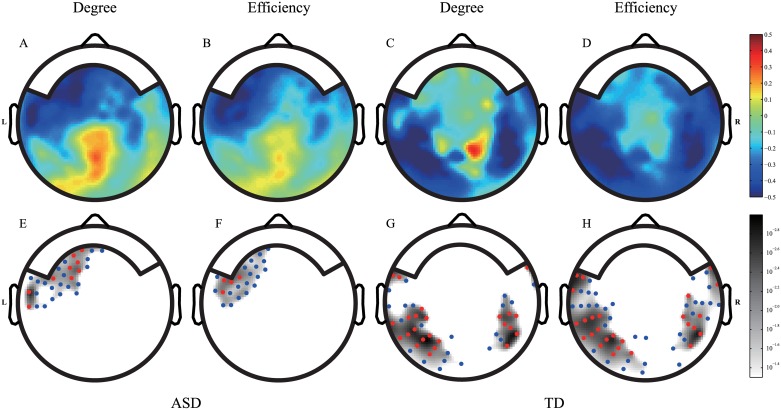
Correlation maps between the nodal level indices and the achievement scale. The network degree was set at 23. Panels (A-D) show the maps of the correlation coefficients between the nodal level indices and the age of the children in the ASD (A, B) and TD (C, D) groups. Panels (A) and (C) show the correlation coefficients between the nodal degree and the achievement scale. Panels (C) and (D) show the correlation coefficients between the nodal efficiency and the achievement scale. The corresponding *p*-value maps (E-H) are shown under each correlation map. Red dots indicate the regions in which the *p*-values of the nodes are less than 0.01. Blue dots indicate the regions in which the *p*-values are between 0.01–0.05.

Fig 7 shows the correlation maps between the nodal level indices and the raw scores of the achievement scale, as well as their corresponding *p*-value maps. In Fig 7A–7D, the color bar ranges from -0.5 to 0.5, with blue indicating negative correlation coefficients and red indicating positive correlation coefficients. Fig 7E–7H shows the *p*-value maps that highlight regions (shaded regions) with significant correlations between the nodal indices and the achievement scores (*p* < 0.05). The sensor locations are indicated by red and blue dots with their corresponding *p*-values. Fig 7A and 7B show that the negative correlation between the nodal level indices and the achievement scale scores was statistically significant in the left temporal and left frontal areas of the ASD group. The regions that showed negative correlations were similar in terms of the nodal degree and nodal efficiency. Negative correlations were also observed in the left temporal-occipital area and right temporal-parietal area of the TD group. Therefore, although NDPs were found in both the ASD and TD groups, but they revealed differentially developing regions.

We did not find an NDP between the nodal level indices and age in the ASD group (S5 Fig). The correlation coefficients between the nodal level indices and age were approximately zero across the whole brain in the ASD group. In the TD group, NDPs were observed between the nodal indices and age across the left temporal-occipital area and right temporal-parietal area.

## Discussion

### Complex auditory and visual stimuli

The main reason for presenting a video program to participants was to ensure that the children were conscious. The level to which the participants focused on the program was not evaluated. One child with ASD did not select an animated movie but instead selected a program with static pictures. Therefore, there were few differences between the groups with regard to the visual information during MEG recording. The stimuli that were presented to participants included narrative auditory information and visual processing information. Compared with static figures and pure-tone stimuli, the stimuli in our experiment were complex dynamic multidimensional stimuli [39]. To comprehend the stories in the program, participants needed to integrate sensory information from different modalities. Language processing and comprehension, attention, visual processing, and appropriate retention of visual memories were involved during the complex video presentation. Therefore, it could be expected that the entire brain was active during the procedure. Although it is difficult to specify the neural response to complex stimuli during the experiments, we studied the network development in the brain based on the age and achievement scale, which are not indices of specific brain function but are general indicators of brain development. Therefore, complex stimuli that induce reactions in the entire brain are more suitable than simple stimuli with reactions only in specific brain regions. Moreover, in the Watts-Strogatz model, the nervous system is modeled as a complex network and evaluates the properties of the entire network across all subgraphs. The nodal degree and efficiency tend to describe the importance of a node according to its relationship with the rest of the nodes in the network.

### NDPs of children with ASD at the level of nodes

The nodal level NDPs in children with ASD were the key finding of this study. There were obvious differences in the NDPs of the two groups at the nodal level. Nodal level mapping revealed that NDPs were located in different regions in the children with ASD and the TD children. Gogtay et al. [40] suggested that typical early brain development occured from back to front, which corresponded to the NDPs of the temporal and occipital areas observed in TD children. The early development of children with ASD started from front to back, i.e. from the frontal and temporal lobes [20]. Our functional data are, to some degree, supportive of these anatomical findings regarding the early sequence of development.

The degrees of the nodes were significantly associated with age and the achievement scale scores in the TD group. The NDPs of the nodal indices and age (S5C and S5D Fig) were similar to the NDPs of the nodal indices and the achievement scale scores (Fig 7C and 7D) in the TD group. These results were predicted because age and the achievement scores were highly correlated in the TD group (Pearson correlation coefficient, 0.8408). The nodal degree and the achievement scores were positively correlated in the parietal lobe. The locations of the visual stimuli and the representations of a visual scene are processed by the parietal lobe in humans [41]. Yan et al. [42] have proposed that the parietal regions act as a visual information exchange hub in the brain. Therefore, high achievement scores in TD children may rebalance their brain connections in the parietal lobe for visual processing and information exchange and reduce connections in other sensory areas. The nodal degrees and efficiencies of the left temporal-occipital area and right temporal-parietal area decreased with improvements in the achievement scale raw scores. The achievement scale is related to crystallized intelligence, and TD children with high crystallized intelligence may not need to maintain redundant connections to transmit new information in the left temporal-occipital and right temporal-parietal areas.

The correlation maps of the nodal indices and age showed different patterns compared with the maps of the nodal indices and the achievement scale scores in the ASD group. These results may be related to the developmental delay in children with ASD. The Pearson correlation coefficient between age and the achievement scores was 0.6313 in the ASD group, which was significantly lower than that in the TD group (difference: *z* = -1.73, *p* = 0.0418 in one-tailed test). Due to the atypical development in children with ASD, we did not find NDPs in the nodal indices and age correlation maps. However, Fig 7A and 7B revealed that the nodal level indices were related to the development index. Therefore, we suggest that the atypical developmental trajectory of children with ASD is directly related to the development of the achievement scale rather than age. The nodal indices in the frontal area decreased as the scores on the achievement scale increased, a phenomenon we have termed negative development. Our results are consistent with several studies, including an fMRI study by Lee et al. [43], which found that the connections in the frontal cortices changed with age in children with ASD. In our previous work [26], a negative correlation between the power of gamma oscillation and mental processing scale was also located in the left frontal area, i.e., the same area that showed negative NDPs in the present study. Therefore, the concept of development patterns is not limited to the graph theory method. Courchesne et al. [21] demonstrated that the frontal areas of children with ASD had the highest level of overgrowth at a particular age. In an EEG study of infants with high-risk of ASD, Orekhova et al. [44] reported that the electrodes demonstrating hyper-connectivity were located in the left fronto-central region. They also reported a tendency of higher connectivity in the left anterior area. Other studies have shown that participants with ASD usually demonstrate hypo-connectivity in later life [18, 45, 46]. We suggested that the negative NDPs on the nodal level of the left frontal area reflect the developmental trajectory of hyper-connectivity in infant high-risk of ASD or children with ASD during toddlerhood to the hypo-connectivity in later adolescence and adulthood. This change in the frontal areas may account for the deficits in social skills that are typically observed in children with ASD. Children with ASD typically recruit more neural areas to perform a task. Kana et al. [47] reported that language and imagery are not well integrated in children with ASD, and that they use visualization to support their comprehension of language. Therefore, the finding that the sensory lobes of the TD group showed negative development, which was not seen in the ASD group, is reasonable given that children with ASD typically recruit more sensory areas during task performance. The redundant connections of sensory areas where the nodal level indices were reduced in the TD group may lead to sensory overload in later life. One outcome of these two different developmental trajectories is the significant difference in the nodal efficiency in the inferior border of the right temporal lobe between children with ASD and TD children (Fig 6). The higher medians of the nodal efficiency of the ASD group reflected greater information flow and processing in those sensory areas. Taking this one step further, it may relate to the new criterion for diagnosis in DSM-5, i.e. the unusual interests in the sensory aspects of the environment. Atypical development of sensory areas was incorporated with NDPs in the frontal area. Our results support the viewpoint of Hilton et al. [48], that sensory responsiveness is a predictor of social dysfunction severity in high functioning ASD. Sensory overload may also be associated with memory performance in ASD. In an online questionnaire study of ASD, memories of sensory details were reported to be related to the age at which the earliest memory occurred in children with ASD [49]. The nodal level differences between the two groups were observed in the right temporal area. The ASD group showed higher network efficiency than the TD group. Strong NDPs were observed in the right temporal area in the TD group. Therefore, these group differences were the outcome of gradual changes in the TD group during development. Previously, we reported a rightward connectivity between parietotemporal areas of children with ASD [26]. Here, we suggested that one reason underlying this rightward connectivity is that children with ASD lose the typical negative NDPs in the right temporal area.

**Fig 8:**
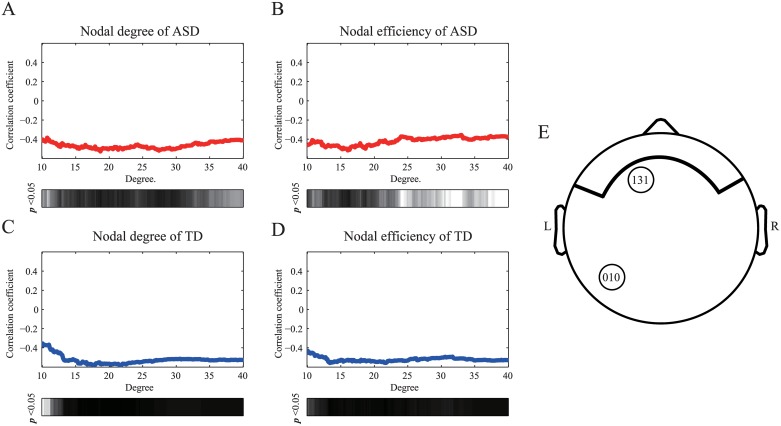
The correlation coefficients between the nodal level indices for two typical sensors and the raw scores of the achievement scale as a function of degree. Panels (A) and (B) show the NDPs of the ASD group on the sensor 131. The NDPs of the TD group at sensor 10 are shown in panels (C) and (D). A strong negative correlation can be observed in both groups. The shaded bars indicate *p*-values less than 0.05. The sensor locations are shown in panel (E).

Based on our results, it could be argued that the NDPs at the nodal level observed in this study may only be found when the network degree is 23. To confirm the NDPs of the two groups at different network densities, we examined the association between nodal indices for two typical sensors, namely sensor 10 for the TD group and sensor 131 for the ASD group, and the achievement scale across different network degrees (Fig 8). The NDPs of the ASD group in anterior brain areas and the NDPs of the TD group in posterior brain areas could be found across different network densities.

### Atypical network development at the network level

Our results at the network level indicated that the ASD group exhibited signs of atypical network development. Figs 4 and 5 indicated that TD children with high scores on the achievement scale adjusted their brain networks to exhibit strong local clustering during the task; this was not the case in the ASD group. Small-world networks exhibit the ability to balance global and local efficiency. Therefore, brains with effective networks may employ a strong small-world structure to process “noisy” information through the whole brain during the resting state. Velázquez et al. [50] proposed that individuals with ASD withdraw into their inner world because they are processing more information than individuals who do not have a diagnosis of ASD. Therefore, in children with ASD, functional networks may maintain strong small-world organization in order to process “noisy” information, including during a visual processing task.

Results of the Shapiro-Wilk test (S2 Fig) suggested that the normality of indices may also be related to the NDPs at the network level. The test statistics of *L* were high in the range of the average degree from 15 to 25 (S2A–S2C Fig). The null hypothesis that the *L* values of both groups were normally distributed cannot be rejected concerning the NDPs of TD children. The normality of the graph index might be an alternative method to aid in the decision of the proper network density for parametric analysis; however, the average degree for nodal level analysis was chosen from where the NDPs were found at a network level.

### Why the network level indices are similar

We have previously suggested that the brain network of a well-functioning brain possesses a stronger ability to direct network connections to local areas during the working state [23]. We have defined a well-functioning brain network as that observed in TD children. As presented in Fig 2C, the specific edges in TD children were mainly short-distance connections within local areas. In contrast, the specific edges in children with ASD were mainly long-distance connections. Therefore, TD children likely perform more local adjustments in their brain networks compared with children with ASD.

In most clinical studies, the small-worldness of the control group was usually stronger than that of the clinical group [6, 15, 16]. In a study of adults with ASD, differences in graph indices were observed between the ASD and TD groups [18]. Another study in adults also reported significant differences in the *C* and *L* values of delta band networks between participant with ASD and controls [15]. In an EEG study, Boersma et al. [12] also reported excessive connections in the broad-band network of toddlers with autism, which is similar to our results. They also made measurements in participants watching videos. In contrast to our results, they reported weaker small-worldness in toddlers with autism than in healthy controls [12]. We expected that the *C* and *L* values might present different patterns between the two groups. However, as shown in Fig 3, there was no significant difference in *C* or *L* values between children with ASD and TD children. Several factors may account for these inconsistencies between our results and those of previous studies. The experimental task, which involved viewing a video, may have affected the results. As we have previously reported on TD children [23], the brain network reorganized with a structure closer to regular networks, a negative NDP of small-worldness, in order for processing to occur during the video viewing task. A similar phenomenon of a decrease in small-worldness has been reported in an EEG study of patients with schizophrenia during both resting state and a working memory task [6]. The results indicated that the difference between the groups in the indices of the Watts-Strogatz model on the alpha2 band was more profound during the resting state, but weaker during the working memory task. In the current study, the relationship between the cognitive index and the network level indices was lost in the ASD group. These results suggested that children with ASD have reduced abilities to adjust their brain network compared with TD children. The combination of these contributing factors may have resulted in similar *C* and *L* values in the ASD and TD groups in our study. In the study by Boersma et al. [12], the TD toddlers were at an earlier developmental stage compared with the TDs in our study, and possibly possessed brain networks with stronger small-worldness than our TDs as the extension of the negative development of small-worldness. If the small-worldness of individuals with autism did not change during the toddler-to-pre-school stage, because the development pattern of small-worldness did not exist during this stage in the ASD group, our results may not completely contradict theirs.

### Why we considered the NDPs

The results on the functional connectivity in ASD are currently controversial [19, 51]. The abnormal neural couplings change during the development of the nervous system. The methods in the field of connectivity analysis have not yet reached a consensus concerning of the best approach [52]. However, direct comparison of neural indices between individuals with ASD and age-matched as well as function-matched peers is commonly performed by researchers. In the context of a developmental disorder under a period of rapid development, the neural patterns of children with ASD change with an atypical trajectory. The results obtained by direct comparison may change due to differences in the age range and cognitive function. Several studies have reached similar conclusions, i.e., cognitive performance is associated with neural connectivity in TD participant. Therefore we should also consider the association of functional development with connectivity in clinical participants [19, 53]. Schwartz et al. [19] claimed that including participants with a wide age range when performing a comparison made it difficult to probe the developmental trajectory. This is because studies typically attempt to examine the developmental trajectory by seeking out the difference between groups at different developmental stages. In our study, the NDPs were obtained by investigating the relevance between network indices and development indices. The development indices explicitly define the participants’ developmental stages. Indeed, the NDPs demonstrate the development trajectory of the participants of each group. As a result, we could identify the atypical development trajectory by comparing the NDPs of the clinical and control groups.

### Electromagnetic field spread problem and association measurements

It is difficult to avoid the issue of electromagnetic field spread in the MEG data in brain graph studies. This issue is caused by the following phenomenon: magnetic fields of electrical activity of a certain neuronal source can spread to multiple sensors, thus resulting in the sensors also recording activities from multiple sources. With electromagnetic field spread, signals measured at the sensor level are not independent of each other. The field spread can generate false coherence and dependence between sensors. There are many metrics to measure the association [54]. Most of the association measurements, directed, linear, or nonlinear, can be affected by the field spread problem [52]. Each method has its own advantage and disadvantage. Several studies reported that most linear and nonlinear association measurements work equally well on sensor and electrode brain data [51, 55, 56]. These findings make it difficult to reach consensus on the recommended metric in the brain graph field [52, 57]. Bastos et al. [57] also summarized other strategies to reduce the effect of electromagnetic field spread, including performing source level study by estimating the underlying sources, or employing well-controlled experimental contrasts to subtract the spurious estimation. However, none of the applications could fully eliminate the effects of the electromagnetic field spread problem [57].

The instantaneous property of electromagnetic field spread led to the development of several methods, e.g., the phase lag index [58] and the imaginary part of the coherency [59], which discard the contributions of coupling without any phase lag or difference. Because the field spread of a dominated rhythmic source could be discarded by those methods [57], they have the potential to result in good performance for the studies with a priori assumption, which can lead to a certain band of interests. Transfer entropy and delay mutual information are suitable and promising tools in finding task-relevant changes in functional connectivity [60, 61]; this is because the proper time delays of specific neural functions are easy to be obtained in task-related studies. However, in a study with a complex stimulus, determining appropriate time delay of each sensor-pair is challenging due to the lack of task-relevant prior knowledge.

Mutual information is a simple and generalized model-free approach that may be suitable for analyzing broad band MEG data without a task-relevant prior assumption. We should point out, however, that the mutual information approach, which does not consider the phase difference and time delay between sensors, can easily be affected by the field spread problem. The results of association measurements without time delay and phase difference should be interpreted very carefully. Matlis et al. [51] performed network inference by the cross correlation, and by the weighted phase lag index [62], i.e., a method which is less affected by electromagnetic field spread, to reproduce a part of the cross correlation results. In this study, authors improved the reliability of the cross correlation by alternative association measurement. We investigated three nonlinear methods of coupling analysis, i.e., mutual information, robust interdependence measure (RIM) [18], and synchronization likelihood [63], to estimate the network structure of selected channels on two sets of MEG data of a dataset of TD children as a pre-research. We observed similar patterns in the network structure under visual inspection (S6 Fig).

### Limitations

The present study has several limitations. First, it was difficult to apply a proper correction method for multiple tests. The studies of brain graphs merely applied corrections for multiple testing [16, 23, 31, 64]. At the network level, the optimal network density was found by probing the significance of correlations across all possible network degrees. The small step size of the network degree can provide a distinct variation in the trend of the correlation across different network densities. However, it can also increase the number of tests and reduce sensitivity when using common correction methods, e.g., Bonferroni correction. The reason for this is that the multiple tests at the network and nodal level in the present study are strongly related. The significance and effect size of a certain network density was strongly related to the nearby network density. The results of any one given node and its nearby nodes were also related, thus explain why NDPs were observed over a region rather than a point. Second, the use of data from different individuals to simulate the developmental trajectory of children with ASD and TD children was not optimal. A longitudinal study on several typical participants may provide more reliable data that is easy to interpret, although it may be difficult to perform. Third, the slight difference between the groups in the Riddle sub-test possibly influenced the results. The results from the Expressive vocabulary sub-test indicated that the ASD and TD groups had almost equal verbal abilities. However, the Riddle sub-test may underestimate the intellectual performance of children with ASD. In the Riddle sub-test, the participants need to explore what the examiners are implying; thus, the results are influenced by the limited social interaction ability of children with ASD. Fourth, alternative association measurement, e.g., RIM, needs to be considered in future studies. RIM showed better specificity and sensitivity than mutual information in biomarker extraction [18], and may thus be less affected by the field spread problem because of the estimation procedure of time-delay-embedding.

## Conclusion

We examined the functional networks in children with ASD and in TD children in the context of network development using MEG data. The NDPs, i.e., the relationships between the network and developmental indices, were analyzed both at the levels of the network and node. The results at the network level demonstrated that children with ASD exhibited atypical network development and possessed less ability to rebalance local and global connections during a video viewing task. In this study, the key finding was that the NDPs of the ASD and TD groups, which were obtained from the raw scores of the achievement scale and nodal level indices, were located in different areas at the nodal level, which implied an altered developmental network trajectory. The regional network development indicated that children with ASD had negative development in the left frontal area. Compared with children with ASD, TD children were able to balance their networks between the hub area (parietal area) and sensory areas (left temporal-occipital and right parietal-temporal areas). Our functional data were compatible with the regional sequence of early brain development in anatomical studies of ASD and TD [20, 40]. Furthermore, our findings indicate that the atypical development of functional networks in ASD is related to the achievement scale scores rather than age. It is possible that information processing strategies were different between the TD and ASD groups in our experiment. The weak connections in the sensory areas of TD children with high achievement scores indicate efficient filtering of useless information, since the TD children with high achievement scores had more crystallized knowledge. The tendency of children with ASD to gather detailed information resulted in less involvement of the frontal areas, but more involvement of the sensory areas. Children with ASD who had high achievement scores may have higher information processing abilities in the sensory areas.

## Supporting information

S1 Fig Results of the Shapiro-Wilk test on elements of the association matrix. Panels A-C show the test statistics of the association matrix of the ASD group, TD group, and both groups; the corresponding p-values for each test are shown in D-F, respectively. The elements are indicated by a gray color when the p-values are less than 0.05.
(EPS)

S2 Fig Results of the Shapiro-Wilk test on network level indices. Panels A-C show the test statistics of the characteristic path lengths of the ASD, TD, and both groups as a function of the network degree from 10 to 40. The clustering coefficient results are shown in panels D-F. The shaded bars show the intervals at which the graph indices did not come from normal distributions at a significance level of 0.05.
(EPS)

S3 Fig Results of the normality test on nodal degree. The test statistics of the degree of the node when the average degree was 23 in the ASD (A), TD (B), and both groups (C). The regions that did not follow a Gaussian distribution (*p* < 0.05) are shown in the *p*-value maps (D–F).
(EPS)

S4 Fig Results of the normality test on nodal efficiency. The test statistics of the efficiency of the nodes when the average degree was 23 in the ASD (A), TD (B), and both groups (C). The regions that did not follow a Gaussian distribution (*p* < 0.05) are shown in the *p*-value maps (D–F) below the corresponding statistics maps.
(EPS)

S5 Fig Correlation maps between the nodal level indices and age when the network degree was 23. Panels A–D are maps of the correlation coefficients between the nodal level indices and the ages of children in the ASD (A, B) and TD (C, D) groups. Panels A and C show the correlation coefficients between the nodal degree and age in each group. Panels B and D show the correlation coefficients between nodal efficiency and age for each group. The corresponding p-value maps (E and F) are shown under each correlation map. Red dots indicate areas where the p-value of the node is < 0.01, while, blue dots indicate areas where the p-value is between 0.01 and 0.05.
(EPS)

S6 Fig Average functional networks obtained by mutual information (MI), robust interdependence measure (RIM), and synchronization likelihood (SL). Panels A, C, and E are networks of the 3-year-old group. Panels B, D, and F are networks of the 4-year-old group. The network density was set to ensure that all vertices were connected to other parts of the graph. Panels in each row correspond to the networks that were obtained by each method.
(EPS)

S1 Table Participants and stimuli. The characteristics (participant number, group, gender, age, scores on sub-tests, achievement scale) of each participant are provided. The names and the types of videos are also listed.
(XLSX)

S1 File Data set of all network indices for statistical comparisons and results. (ZIP)
