# Correction: Detection of atypical network development patterns in children with autism spectrum disorder using magnetoencephalography

**DOI:** 10.1371/journal.pone.0188813

**Published:** 2017-11-27

**Authors:** The PLOS ONE Staff

There is an error in the Funding section. The correct funding information is as follows: This work was partially supported by JST CREST, Grant Number JPMJCR14D2, Japan and the Core Research for Evolutional Science and Technology and the Center of Innovation Program, the Japan Science and Technology Agency. This work was also partially supported by Japan Science and Technology Agency KAKENHI Grant Number 15H05707. The funders had no role in study design, data collection and analysis, decision to publish, or preparation of the manuscript. The publisher apologizes for the error.
